# Supplementary material for: Role for the shelterin protein TRF2 in human herpesvirus 6A/B chromosomal integration
Source: PLoS Pathog. 2020 Apr 22;16(4):e1008496. doi: 10.1371/journal.ppat.1008496 (PMC7197865; doi:10.1371/journal.ppat.1008496)
Supplement: Attachment — Submitted filename: Response to reviewers-final.docx [file ppat.1008496.s001.docx]

**Part II – Major Issues: Key Experiments Required for Acceptance**


Reviewer #1: 1. It is argued that TRF2 is not required for IE2 localization at viral replication compartment. These experiments are based on a system with Dox-inducible shRNA, but unfortunately, these experiments lack certain controls of the Dox system. Dox may have a number of off-target effects, which are not controlled for in the experiments (e.g. Ahler et al., Doxycycline alters metabolism and proliferation of human cell lines. PLoS One, 2013).
To appreciate the data in fig.8, a control for shTRF2 is missing; that is, a control which is also treated with Dox.

The reviewer raises a valid point. To control for this potential issue we included, as an additional control, a control shRNA treated with Dox (figure 7B). These new experiments exclude any off-target effect attributable to Dox and confirmed our previous results.

In fig.8D it is argued that 53BP1 is located in foci in the absence of TRF2. It may be correct, but Fig.8A shows that TRF2 is indeed expressed in the presence of Dox, and since there is no staining for TRF2 in Fig. 8D it is difficult to draw this conclusion.

Also, there seems to be an inconsistency between the expression level of TRF2+Dox in Fig.8A and the level found by immunofluorescence in Fig.8B, which raises the issue of the kinetics of TRF2 expression during Dox treatment.

The experiments were repeated several times and gave consistent results. However, given the complexity of the DDR response pathway and since this is not the main focus of our study, we have decided to remove these data at this stage.

2. In the title the authors claim that recruitment of TRF2 prevents DNA-damage response, but the investigation of this response is rather superficial and does not provide much inside into what is actually going on. It is speculated in the discussion that integration occurs through HR. Nevertheless, the authors restrict their investigation to only examine the expression of a phosphorylated 53BP1 by immunofluorescence, although 53BP1 in association with RIF1 (not examined) are known for promoting NHEJ repair. Indeed 53BP1-RIF1 may antagonize BRCA1 (not examined) and HR. A lot is known about these pathways, and it should be feasible to investigate them in much greater details to provide a better understanding of how viral integration occurs and how HHV-6A/B interferes with the DNA-damage response.

We agree with the reviewer that we are only scratch the surface of the DNA damage response triggered during infection. As discussed above, characterization of this pathway goes beyond the scope of this manuscript and will require additional experiments. As addressed above we therefore removed the DDR data from the paper and replaced it with new data indicating that in the absence of TRF2, the viral IE2 protein no longer localizes at cellular telomeres. These new results are presented in figure 7.

Other points:
3. Fig. 3B: The exposure of the Alu-probed blot should be similar to the anti-TRF2 with (TTAGGG)3 probe to evaluate the data, in particular since nothing is provided about how linearity in the exposure interval is verified. One drawback of dot blots is the lack of size separation and thus an accumulated signal from everything that may bind the probe.

The blots shown are exposed for the same amount of time and all within a linear range. All spots were analyzed by densitomery. For calculations, we used signals that were within the linear range and not saturated.

The HHV-6 DR6 probe apparently binds something in anti-Ig immunoprecipitated samples. If developed longer, it could look quite similar to anti-TRF2 IP. So how do the authors know that what is recognized in anti-TRF2 is not simply “non-specifically” precipitated DR or something completely different with cross-reactivity to the probe?

Although true that the anti-Ig precipitated some material (for HHV-6A and not HHV-6B), the amount precipitated was always much less than with anti-TRF2. The exposure is the same for both conditions (Ig and TRF2) so increasing the exposure time, would increase the signals in both conditions.

4. Presentation of the kinetics for accumulation of viral DNA copies would make fig. 1D more convincing. What was the MOI for these infections?

The MOI used for infection was 0.1. We have replaced the data (Figures 1 D-E) with a dot blot image showing increased telomeric signals during infection to make it more visual.


5. To appreciate the lack of colocalization of p41 with telomeres (fig.6G), the same 3D imaging as shown for fig. 6E should be provided. Done (Figure 6D).

Reviewer #2: -Fig. 9b, lines 371-381: When using ddPCR, is it possible to specifically measure the “relative HHV-6A/B integration frequency” among a population of U2OS cells that contain either the HHV-6A/B genome integrated or within viral replication compartments? It’s clear that TRF2 depletion leads to a reduction in the relative levels of HHV-6A/B viral genomes following infection of U2OS cells for one month, but is this a decrease in telomere integration or viral DNA replication? Providing quantitative analysis of FISH-IF (HHV-6A/B DNA and TTAGGG probe) on shSCR-U2OS and shTRF2-U2OS cells by counting the number of integration events within the total population of cells would be a potential means of addressing this question.

We agree with the reviewer’s suggestion that counting the integration events by FISH could be used to estimate integration frequency. However, the number of cells containing integrated HHV-6 is relatively low after a month of culture. In a previous paper (Gravel al J Virol 2017), the ddPCR approach and single cell cloning was compared to estimate the integration frequency. Both assays yielded comparable results. We are therefore confident that the reduced integration frequency observed upon TRF2 in knockdown is correct. We added additional information in the materials and methods section to better explain how integration frequency was determined. Furthermore, new results added in (figure 7B-C) indicate that the initial phase of infection is similar between controls and TRF2 knockdown cells.


Reviewer #3: Major issues (I used downloaded TIF versions of the figures.):
1. An important, but readily addressable problem in the paper is that the figures and text do not explicitly state that the confocal microscopic images are of small numbers of nuclei, or of single nuclei. None of the images have scales. In the absence of DAPI staining (e.g., Fig. 1B) it would be helpful to provide outlines of nuclei. The DAPI images in Fig. 7 are too dim to be useful. All nuclei have now been outlined, as suggested.

2. The Fig. 4B y-axes should be something like: “Number of HHV-6A genomes/Number of host genomes”. In Fig. 4C, the number of HHV-6B genomes per cell (~80,000/cell) is extraordinarily high, especially since only 2 of the 5 nuclei shown in Fig. 1C appear to be infected with HHV-6B. Please double-check the math.

The y-axes represented the number of viral copies per ug. Since these results were negative and less relevant, Figure 4 has been removed.

3. In Fig. 5A, it would be helpful to show a merged image. It appears that the two images under IE2+ conditions are not presented at the same scale. The figure has been replaced with more comprehensive images showing IE2 and TRF2 at all kinetic time points. The merge images are also presented, as suggested.

4. The explanation of the Fig. 9 integration frequencies is inadequate. A brief description of the method is needed (not just the reference). It is not clear whether in this context, the assay measures integration frequency, as opposed to the frequency of 30-day survival of cells in which integration occurred. The reviewer makes a valid point and a difficult one to answer. To make it more comprehensible, a more detailed description of the assay has been added in methodology section. Furthermore, a sentence describing the possibility raised by the reviewer has been included to outline the limitations of the study.


5. Why are there so many more IE2 spots in TRF1 and POT1 transfected cells than in non-transfected controls? IE2 is present in numerous donut-like structures in the transfected cells, but not in the non-transfected cells. How might this affect interpretations?

The number of IE2 foci varies from cell to cell and are not influenced by the transfection. We now have selected images with similar numbers of IE2 foci after transfection with TRF1 and empty vector (figure 5B).

**Part III – Minor Issues: Editorial and Data Presentation Modifications**


Reviewer #1: 1. Fig. 8: It needs to be clearly specified when the western blotting for shRNA is performed and when the infection experiment and the immunofluorescence staining is performed to make sure the expression on the Western blot is representative for the expression at the time of immunofluorescence analyses.

Yes, the western blotting was performed at the same time as the IFA. This information has been included.

2. The experimental conditions behind fig. 1E is not clearly described. Although some references to other papers are provided one should be able to understand the concepts from this paper alone. Thus, how was the data adjusted for the fact that uninfected cells keep dividing whereas infected cells stop dividing? How was telomeric repeats estimated?

We have now removed this figure and replaced it with a better understandable dot blot to show increase in telomeric signals during infection.

3. l.61: …that play a role… -> that are required Corrected

4. l.315: “generally used” is an overstatement, as it seems only to refer to the authors themselves. Rephrase to “has been used” Corrected

5. l.505: mead -> mean Corrected

6. l.526: “uninfected (white)” is “uninfeceted (blue)” on my computer?? Corrected

7. l.350: “colocalized perfectly with VRC, as evidenced by IE2 staining” It is not clear what qualifies it to be perfectly. Next, what is actually shown is colocalization to IE2. Therefore, it should be rephrased to “colocalized with IE2” Corrected

8. l.358, l.359 and several other places: It seems that the designation VRC is an interpretation. Better to describe what is actually examined and observed. In fig. 1G HHV-6A delta(Imp TMR) induce pronounced telomeric signals One colocalize very well with IE2, the other not very well – how should this be interpreted? And what does this mean for using IE2 staining as evidence of VRC?

This is a valid point. In our hands, the IE2 antibody is a superior antibody than the anti-P41 antibody and this is why we use it more frequently. P41 is the viral DNA processivity factor that associates with viral DNA during replication. It therefore marks VRC. We have now provided results (figure 1B) to show that during productive HHV-6A infection, IE2 and P41 colocalize. Having demonstrated this, we would argue that IE2 can be used as reliable marker of VRC.

The images in figure 1G were improved to better visualize colocalization between IE2 and telomeric signals.

9. Fig. 6C does not provide additional information to what is already stated in the text. Thus, it is not necessary.

Figure 6C was added at the request of a previous reviewer.

10. Fig. 8C: In my version of the paper, the mock cells and the HHV-6A cells -Dox (which are negative for antibody staining) have also much more dimly DAPI staining. Why is that?
(It may raise concerns about the compensation strategies during the collection of the immunofluorescent pictures.) Figure 8 has been deleted.

11. Fig.4: In knock-down experiments it is concluded that depletion of TRF2 does not affect viral replication. In particular because of the lack of an effect of the depletion, it is not clear whether the remaining amount of TRF2 is sufficient to exert a given function.
Without this, fig. 4 is difficult to interpret and does not add much.

The reviewer makes a valid point. Figure 4 has been deleted.

Reviewer #2: Comments in this passage are focused primarily on the lack of clarity for some of the immunofluorescence images. It’s unknown whether the lack of clarity is attributed to low image resolution or inefficient staining.
-Figs. 1b, 6a, 6b, 6e: Including a DAPI counterstain would more clearly define the location of the cell given that its unknown whether these images contain one cell or multiple cells in the field of view. The nuclei have now been outlined to make the image clearer.
-Fig. 1b, lines 232-236: Given the low image resolution, it’s difficult to differentiate between punctate (cellular) and “splotch” telomere signal. Would suggest providing a higher magnification of each example paired with localization of IE2 to more clearly convey this point. New figures have been added.
-Fig. 6a: Surprising the telomere signal is very low (nearly absent) in mock group for Fig. 6a, but not in Fig. 6b. Furthermore, unable to observe “IE2 is also perfectly colocalized with VRC alongside p41” again due to low fluorescence intensity. May also want to include a comment in text describing the highlighted area containing the white box. This picture was included to show colocalization of P41 with IE2. This figure has been deleted and replaced with figure 1B to show colocalization of P41 and IE2 during productive infection of T cells.
-Figs. 6b, 6f: How many cells were counted? Information is missing from the text. Added to the text of figure 6 legend.
-Figs. 7a, 7b: IE2 signal is very low in “HHV-6A; non-transfected” group. POT1 signal is also very low in the “POT1 transfected” group. New images are now provided. Results of the POT1 experiments have been deleted.
-Fig. 8d: In contrast to “HHV-6A (+) DOX” group, the “patchy” TTAGGG signal is absent from regions of IE2 in the “HHV-6A (-) DOX” group. Moreover, it appears that TTAGGG punctate foci are excluded from the “more pronounced VRC.” This observation appears to be different from other figures in this manuscript.
Figure 8 has been removed.

-Lines 194-95, Fig. 3: Unclear why an input sample was taken prior to sonication. Input should have been from the same pool of chromatin that was used in the TRF2 and IgG ChIP. It’s known that sonication aids in cell lysis during chromatin shearing, therefore the % input used in the figure does not accurately represent the efficiency of the ChIP. Thank you to the reviewer for pointing this out. This was an error during the writing of the methodology section. After verification, the input was actually taken after sonication. The text was modified accordingly.

-Line 199, Fig. 3: State the quantity (µg) of normal rabbit IgG used in the ChIP (not volume). If the same µg of antibody was not used for IgG and TRF2, authors would need to specify reason when representing background signal. The quantity of rabbit IgG (4µg) and anti-TRF2 (4µg) were added to the text.

-Figs. 3c, 3e: If results are from 3 independent experiments, error bars and % input for IgG (background signal) is missing. Including IgG in this figure is critical given the noticeable IgG signal in HHV-6A and HHV-6B infected cells (see Figs. 3b and 3d).

The result of one representative of three independent experiment is shown. This is indicated in the figure legend. The IgG signals have been added, as requested.

-Line 206, Fig. 2c: Why were different amounts of MBP (25 ng) and MBP-TRF2 (50 ng) used in the in vitro binding assay? Puzzling given that MBP alone serves as a control for the nonspecific binding to HHV-6A DNA. The quantity was adjusted in terms of molecules. Since MBP is twice as small as MBP-TRF2, we added twice the amount of MBP-TRF2 to have an equal number of molecules (0.6 pmoles) per well.

-Line 295-296: Consider including RNAPII ChIP data into Fig. 3 or supplementary information rather than stating data is “not shown.” The RNAPolII ChIP data is now presented under figure 3B.

-Fig. 8a: Would suggest re-running this western to achieve individual clean bands with densitometry analysis. The western blots to assess TRF2 knockdown were repeated as suggested (figures 7A and 8A).

-Given the similar roles of TRF1 and TRF2 in the maintenance of telomere stability, may want to include a very brief comment why TRF2 and not TRF1 was the primary focus? Could there be redundancy between both TRF1 and TRF2, such that depleting both proteins or TRF1 alone leads to a greater decrease in viral integration? This is purely speculative…experiments are not necessary. TRF2 was mainly study because of the quality of the anti-TRF2 antibodies available. As suggested, we added a sentence of the potential effects of TRF1 knockdown on integration.

Reviewer #3: Suggested edits or points needing clarifications:
1E – y-axis needs a label – is this intensity or copy number. This figure has been replaced by a dot blot.

Fig. 2. Panels A and B should have competitors expressed in pmoles, as is done for Panel C. Corrected

In Panel A, what are the units for the 10, 100, and 1000 telo and non-telo numbers. Units (pmoles) are now indicated.

In Panel C, what are the molar ratios between the target (25 ng of genomes) and the competitors?- Competition was done using 15x and 30x of competitors. See text for details.

Fig. 4A. It is hard to tell whether the bands seen in the +Dox lane are background expression or leakage from the adjacent lane. If it is the latter, the authors’ case would be stronger. These results have been deleted.

Silencing TRF2 seems to have a small but fairly consistent positive effect on HHV-6A genome replication. These results have been deleted.


Fig. 6A. No scales are provided. Are these high-magnification images of single nuclei? State that the boxes are the region shown in the 3D representation. What do you make of the IE rings (more common with the deletion mutant)? The difference in localization relative to the TTAGGG probe should be mentioned.

The nuclei have now been outlined by a dashed line. The IE rings were observed (both for WT and mutant) as a result of the deconvolution process. The images were redone. Our results indicate that WT and mutant IE2 localize at telomeres with equal frequency.

In 6C, it is easy to see the relationship of the patterns for the TTAGGG, IE2, Merged, and XYZ panels, because they are presented in the same relative positions in all four panels. For the IE2 deletion data in panel 6E, the XYZ panel is rotated relative to the others. I do not see how the XYZ panel in the empty vector row relates to the first three panels. Related to this, I could only figure out how the deletion 3D image relates to the rest in its row. What is the scale? What is the definition of “at telomere”? It would be helpful to show at least two images of the 3D reconstructions: one in an orientation that corresponds to the XYZ panel (which should correspond to the others in that row), and one rotated to make whatever other point is intended. Movies that show the reconstruction rotating in space would be helpful.

The reviewer likely refers to figure 6B (and not 6C as 6C is a graph). The XYZ images were removed. We now provide a 3D image in the same plane as the 2D images. In addition, arrows pointing to IE2 colocalizing with telomeric signals were added to facilitate the viewing. “At telomeres” meant colocalizing with telomeres. We have now replaced “at telomeres” with “with telomeres” throughout the text.

The Fig. 6G legend text should be modified to say “Lack of colocalization between HHV-6A p41 and telomeres in uninfected cells.” Corrected.

Fig. 7A and 7B. In the top rows, why are there so many more IE2+ spots in the 3D rendering? Image 7A was redone without 3D to better show localization of TRF1 at VRC. Depending on the angle used in the 3D rendering, the IE2+ spots are more or less apparent.

Fig. 8B. Why are the TTAGGG signals so much more pixelated than the other signals (looking at the high resolution TIFF images)? A new picture with higher resolution is now included.

Why is the IE2 staining in Fig. 8 panel B so much more diffuse than in Panels C and D. This is not an image size or resolution issue. Figure 8 and is now deleted.

Fig. 9B y-axis: “frequency” Corrected.

line 505. “mean absorbance”-Corrected.
