# Supplementary material for: Role for the shelterin protein TRF2 in human herpesvirus 6A/B chromosomal integration
Source: PLoS Pathog. 2020 Apr 22;16(4):e1008496. doi: 10.1371/journal.ppat.1008496 (PMC7197865; doi:10.1371/journal.ppat.1008496)
Supplement: Attachment — Submitted filename: Editor Letter PLoS revised.docx [file ppat.1008496.s002.docx]

Dear PLoS Pathogens Editors,

Thank you for considering our manuscript for publication in PLoS Pathogens. We are pleased to read that both reviewers were satisfied, for the most part, with our revised manuscript. As requested, we have made the recommended corrections to our paper and provide a point by point response to each comment.

Looking forward to your final editorial decision regarding our manuscript,

Sincerely

Louis

Louis Flamand PhD, MBA

Chair and Professor

Department of Microbiology-Immunology

Faculty of medicine

Laval University

T: 418-525-4444 x46164

Response to reviewers comments.

Reviewer #1

l.35: ”integration frequency” is mentioned twice in the same sentence. Please correct. Correction made.

Reviewer #2

**Part II – Major Issues: Key Experiments Required for Acceptance**

Fig 1b, lines 247-249: Based on the images provided it appears that strong IE2 signal is excluded from the p41 signal, hence the two proteins are adjacent rather than colocalizing. What is the Mander’s colocalization coefficient for p41 and IE2? This is important to establish given the use of IE2 as a marker for viral replication compartment in all subsequent figures. Also, poor image resolution (pixilated) may be a culprit for the lack in clarity.

We apologize if the image appears to be of poor resolution. In our hands, the image looks fine. As requested, we have analyzed several images and provide (line 247) the Mander’s colocalization coefficient for p41 and IE1 (0.92±0.07).

**Part III – Minor Issues: Editorial and Data Presentation Modifications**

Fig 1b, lines 251-253: Based on very low signal “telomeric probe resulted in the detection of many discrete punctate telomeric signal” is not apparent in Fig 1b, but is in Fig 1c. Again, this is the same general issue as brought up in the previous review of low signal or low image resolution.

We agree with the reviewer that it is sometime difficult to observe the discrete punctate telomeric signals, especially in non-infected cells. When acquiring the images, we use settings that are the same for both uninfected and infected cells. Considering that infected cells have much high levels of telomeric signals, visualization of telomeric signal in uninfected cells is suboptimal. However, we felt important to keep the settings the same for all conditions.

-Fig 1g, lines 275-284: In this figure it is obvious that HHV-6A and HHV-6AdeltaimpTMR have an IE2 signal that localizes with telomere. However, without providing a positive IE2 infected cell for HHV-6AdeltaTMR its difficult to conclude “the viral genome were responsible for increased telomeric signals observed.” To make this conclusion an HHV-6AdeltaTMR infected cell with positive IE2 signal would need to be shown with telomere signal that does not colocalize. Poor image resolution maybe a culprit again, but the data presented should be clear.

The HHV-6A delta TMR condition (figure 1g last row) does show IE2+ cells. We apologize if this was not apparent. To facilitate viewing, we have added arrows pointing to IE2 expressing cells. In contrast to WT, infection with the HHV-6A delta TMR mutant does not result in massive, easily identifiable telomeric signals.

-Fig 4a, lines 335-336: Provide the Mander’s colocalization coefficient to conclude “TRF2 appeared to colocalize with IE2.”
Mander’s colocalization coefficient are now provided. The following sentence was added on line 332: Depending on the time of infection, between 38% (24 h post infection) and up to 60% (72h post-infection) of IE2 colocalized with TRF2.

-Fig 5a, lines 564-566: Authors need to define the difference between patchy and punctate IE2 signal with regards to VRC. Is it possible to conclude that “infected cells that do not actively replicate viral DNA with TRF2 and IE2 colocalization” when there are no viral DNA FISH controls to support these conclusions of which IE2 staining pattern (patchy vs punctate) represents VRCs?

The reviewer is correct in mentioning that without viral DNA FISH, we cannot absolutely be certain. However, the fact that we do observe diffuse telomeric signal overlapping with IE2, does suggest that these are VRC. We have now modified the text (line341) to the following: As shown in figure 5A (middle row), some cells display large patchy IE2 staining similar to what is observed during productive infection of T cells (Figures 1B-C) and likely representing VRC. Such IE2 patches overlapped with diffuse TRF2 and telomeric signals.

We also modified the legend as follow: The panels in the middle row show images of cells with IE2 patches overlapping with large, diffuse TRF2 and telomeric staining (rectangles). The panels in the third row represent infected cells with punctate IE2 pattern colocalizing with TRF2 and telomeres (dashed squares).

-Fig 5b, lines 346-351: The % colocalization of punctate IE2 with TRF2 was provided in Fig 5a, but this information is missing for IE2 and TRF1.

The % of IE2 localizing with TRF1 (78% ±20%) is now provided (line 353).

-Fig 6d, lines 363-365: The p41 signal appears to be localized as a concentric ring around the nucleus and not “p41 exhibit a diffuse nuclear distribution.”

We agree. The text (line 366) was modified to: As shown in figure 6D, ectopically-expressed p41 exhibited mostly a perinuclear distribution that did not colocalize with cellular telomeres.

-Lines 589-590, Fig 7c: Describe how the percent of IE2 positive cells were “estimated” as opposed to providing an absolute count instead?

The text of legend to figure 7 (line 591) was modified as follows: The percentage of HHV-6A infected cells (from B) was estimated after counting a minimum of 700 cells and scoring the IE2^+^ ones. Results are expressed as mean %IE2^+^ cells ± SD.
